# Outcomes of patients with schizophrenia who discontinued long‐acting injectable antipsychotic therapy due to adverse events: A chart review

**DOI:** 10.1002/npr2.12192

**Published:** 2021-07-01

**Authors:** Taro Kishi, Kenji Sakuma, Makoto Okuya, Masakazu Hatano, Nakao Iwata

**Affiliations:** ^1^ Department of Psychiatry Fujita Health University School of Medicine Toyoake Japan; ^2^ Department of Clinical Pharmacy Fujita Health University School of Medicine Toyoake Japan

**Keywords:** adverse events, long‐acting injectable second‐generation antipsychotic, schizophrenia

## Abstract

**Aim:**

We conducted a chart review to investigate the detailed outcomes of patients with schizophrenia who discontinued long‐acting injectable second‐generation antipsychotic (LAI‐SGA) therapy due to adverse events (AEs).

**Methods:**

The study included patients with schizophrenia and related psychotic disorders who commenced LAI‐SGA therapy between January/1//2009 and March/31/2020 at Fujita Health University Hospital in Toyoake, Japan.

**Results:**

We conducted a chart review of 157 patients with schizophrenia. At the time of this survey, 4 (6.9%), 5 (12.2%), and 10 (17.2%) of the patients in the aripiprazole once monthly, paliperidone palmitate, and risperidone‐LAI groups, respectively, discontinued due to AEs since the start of LAI‐SGA therapy. Three patients required hospitalization for AE treatment.

**Conclusion:**

The severity of these AEs in most patients is moderate (ie, no hospital treatment required). Due to the small sample size, a larger study is needed to confirm/replicate our study results.

## 1 INTRODUCTION

Our retrospective chart review study reported that 39 (24.8%) of the 157 study patients discontinued their long‐acting injectable second‐generation antipsychotic [LAI‐SGA] therapy aripiprazole once monthly (AOM, n = 58), paliperidone palmitate (PP, n = 41), and risperidone (RIS‐LAI, n = 58) within 6 months of starting.^1^ Eleven (7.0%) patients discontinued due to adverse events (AEs). LAI‐SGAs, therefore, appear to have favorable acceptability and tolerability for patients with schizophrenia in clinical practice. However, a cross‐sectional study reported that more than 50% of psychiatrists believed that the AEs are more severe for LAI antipsychotics than for oral SGAs.^2^ The recent review article investigating psychiatrists' attitudes toward LAI antipsychotics reported that although most psychiatrists believed that LAI antipsychotics are proven to prevent schizophrenia relapse compared with oral antipsychotics, some psychiatrists are reluctant to use LAI antipsychotics due to concerns about the safety of LAI antipsychotics.^3^ Therefore, we conducted an additional chart review to investigate the detailed outcomes of patients with schizophrenia who discontinued LAI‐SGA therapy due to AEs.

## 2 METHODS

The study included patients with schizophrenia and related psychotic disorders who commenced LAI‐SGA therapy between January/1//2009 and March/31//2020 at Fujita Health University Hospital in Toyoake, Japan. This study focused on the patients in the AOM, PP, and RIS‐LAI groups who discontinued the therapy due to AEs between the start of therapy and the time of this survey. The final chart review was performed on May/2//2021. This study was approved by the Institutional Review Board of Fujita Health University. Informed consent was obtained via an opt‐out form on the website and in‐hospital bulletin board. Patients with unknown start times for LAI‐SGA therapy or who declined to participate were excluded. The age at the start of therapy, age at AE onset, sex, therapy duration, and treatment status were collected. If there was more than one reason, we confirmed the most appropriate reason with the patients’ primary doctors or selected the most relevant reason.

## 3 RESULTS

We conducted a chart review of 157 patients with schizophrenia. At the time of this survey, 4 (6.9%), 5 (12.2%), and 10 (17.2%) of the patients in the AOM, PP, and RIS‐LAI groups, respectively, discontinued due to AEs since the start of LAI‐SGA therapy. Table 1 lists the patients' characteristics and their outcomes after discontinuing LAI‐SGA therapy. One patient in the AOM group (rhabdomyolysis) and 2 in the RIS‐LAI group (both were akathisia) required hospitalization for AE treatment. Given that the patient with rhabdomyolysis was associated with severe dehydration and severe respiratory infection, the association between rhabdomyolysis and AOM is unclear. This patient did not meet 3 major diagnostic criteria for neuroleptic malignant syndrome^4^, ^5^, ^6^ and was, therefore, not administered dantrolene. With fluid replacement, the patient recovered in approximately 1 week. Because the patient had poor medication adherence, the patient was administered injectable RIS‐LAI after confirming tolerability with oral RIS for approximately 2 weeks. One patient with severe akathisia was administered injectable RIS‐LAI without confirming tolerability for oral RIS. The patient was administered anticholinergic agents after admission, and the symptoms improved within a few days. Another patient's akathisia symptoms were difficult to distinguish from agitation. Although the patient was administered an anticholinergic agent after admission, the akathisia symptoms did not notably improve. The patient refused oral or injectable antipsychotic treatment and was transferred to another hospital within a few days after admission to our hospital. The AEs in the other patients required no hospital treatment. The other AOM patients’ reasons for discontinuation were extrapyramidal symptoms (n = 1) and injection‐site pain (n = 2). The PP patients’ reasons for discontinuation were akathisia (n = 1), fatigue (n = 1), weight gain (n = 2), and injection‐site pain (n = 1). The other RIS‐LAI patients’ reasons for discontinuation were extrapyramidal symptoms (n = 1), akathisia (n = 1), fatigue (n = 1), hyperprolactinemia (n = 3), and injection‐site pain (n = 2). Nine of the 19 patients had their medication replaced with other LAI‐SGAs. The patients who discontinued LAI‐SGA due to injection‐site pain were returned to the oral medication that they took before the LAI‐SGA therapy.

**TABLE 1:** The characteristics of the patients who discontinued long‐acting injectable antipsychotic therapy due to adverse events and their outcomes after discontinuation

LAI‐SGA	Final dose	Age at onset	Age at which LAI‐ SGA started	Sex	Duration of LAI‐SGA treatment	Symptoms	Grade* (treatment status)	Concomitant AP use	Concomitant BENZ use	Duration oral AP before the start of LAI‐SGA	Outcome
AOM	400 mg	41	67	F	7 m	Extrapyramidal symptoms	Mo (Out)	No	No	OARI 12 mg for 6 m	Add anticholinergic agent. Replace with OARI.
AOM	400 mg	39	57	F	4 m	Rhabdomyolysis	Se (In)	No	No	OARI 24 mg for 6 m	Discontinued AP for 1 m. Replaced with RIS‐LAI after using ORIS for 2 m.
AOM	400 mg	22	41	M	19 m	Injection‐site pain	Mo (Out)	No	No	OARI 24 mg for 28 m	Replace with OARI.
AOM	400 mg	24	47	F	3 m	Injection‐site pain	Mo (Out)	Yes	Yes	OARI 30 mg for 12 m	Replace with OARI.
PP	100 mg	52	77	M	2 m	Akathisia	Mo (Out)	No	Yes	none	Add anticholinergic agent. Replaced with RIS‐LAI after using ORIS for 6 m.
PP	150 mg	34	46	F	1 m	Fatigue	Mo (Out)	Yes	Yes	OPAL 9 mg for 24 m	Replaced with AOM after using OARI for 6 m.
PP	50 mg	40	41	F	20 m	Weight gain	Mo (Out)	No	No	OPAL 6 mg for 3 m	Replaced with AOM after using OARI for 6 m.
PP	150 mg	26	45	M	5 m	Weight gain	Mo (Out)	No	No	OPAL 9 mg for 2 m	Replaced with AOM after using OARI for 3 m.
PP	150 mg	18	24	F	1 m	Injection‐site pain	Mo (Out)	No	Yes	OPAL 9 mg for 6 m	Replace with OPAL.
RIS‐LAI	25 mg	26	30	F	3 m	Extrapyramidal symptoms	Mo (Out)	Yes	Yes	ORIS 3 mg for 12 m	Add anticholinergic agent. Replace with ORIS.
RIS‐LAI	37.5 mg	23	27	M	1 m	Akathisia	Se (In)	No	No	ORIS 2 mg for 48 m	Add anticholinergic agent. Transferred before restarting any AP.
RIS‐LAI	25 mg	46	56	F	7 m	Akathisia	Mo (Out)	No	Yes	ORIS 2 mg for 48 m	Add anticholinergic agent. Replace with ORIS.
RIS‐LAI	25 mg	25	55	M	3 m	Akathisia	Se (In)	No	No	none	Add anticholinergic agent. Replaced with AOM after using OARI for 6 m.
RIS‐LAI	50 mg	15	15	M	17 m	Fatigue	Mo (Out)	Yes	No	ORIS 4 mg for 4 m	Replaced with CLO.
RIS‐LAI	25 mg	27	27	F	7 m	Hyperprolactinemia	Mo (Out)	Yes	Yes	ORIS 3 mg for 12 m	Replaced with AOM after using OARI for 6 m.
RIS‐LAI	37.5 mg	28	37	F	14 m	Hyperprolactinemia	Mo (Out)	No	Yes	ORIS 2 mg for 48 m	Replaced with AOM after using OARI for 5 m.
RIS‐LAI	50 mg	23	35	F	79 m	Hyperprolactinemia	Mo (Out)	No	Yes	ORIS 2 mg for 12 m	Replaced with AOM after using OARI for 3 m.
RIS‐LAI	25 mg	18	45	F	3 m	Injection‐site pain	Mo (Out)	No	Yes	ORIS 4 mg for 2 m	Replace with ORIS.
RIS‐LAI	25 mg	29	35	F	2 m	Injection‐site pain	Mo (Out)	No	No	ORIS 4 mg for 48 m	Replace with ORIS.

Abbreviations: AOM, aripiprazole once monthly, AP, antipsychotic, OARI, oral aripiprazole, BENZ, benzodiazepin, CLO, clozapine, F, female, In, inpatients status, LAI, long‐acting injectable, M, male, m, month, Mo, moderate, Out, outpatient status, OPAL, oral paliperidone, PP, paliperidone palmitate, (O)RIS, (oral) risperidone, Se, severe, SGA, second‐generation antipsychotic.

* Moderate; discomfort enough to cause interference with usual activity and may warrant investigation. Severe: incapacitating with inability to do usual activities or significantly affect clinical status, and warrants intervention. We referenced the following information (1) FDA (https://www.fda.gov/) and (2).

## 4 DISCUSSION

Our study suggests that although LAI‐SGAs have a risk of AEs, the severity of these AEs in most patients is moderate (ie, no hospital treatment required). There were several limitations to this study. Due to the small sample size, we did not perform a statistical analysis to examine the association between AE incidence/severity and the clinical factors. A larger study is needed to confirm/replicate our study results. Because a recent network meta‐analysis showed that a 3‐month PP formulation prevented schizophrenia relapse,^7^ the effectiveness of this LAI‐AP should be confirmed in clinical practice.

## CONFLICTS OF INTEREST

Dr Kishi, Dr Sakuma, Dr Okuya, Dr Hatano, and Dr Iwata declare that they have no direct conflicts of interest relevant to this study. Dr Kishi received speaker's honoraria from Daiichi Sankyo, Dainippon Sumitomo, Eisai, Janssen, Otsuka, Meiji, Mochida, MSD, and Tanabe‐Mitsubishi (Yoshitomi), as well as research grants from the Japanese Ministry of Health, Labour, and Welfare, a MEXT/JSPS KAKENHI grant (number 19K08082), and Fujita Health University School of Medicine. This work was supported by a MEXT/JSPS KAKENHI grant (number 19K08082). This funding source had no role in the design of this study and will not have any role during its execution, analyses, interpretation of the data, or decision to submit results. Dr Sakuma has received speaker's honoraria from Eisai, Kissei, Meiji, Otsuka, and Torii and has received a Fujita Health University School of Medicine research grant, as well as a MEXT/JSPS KAKENHI Grant for Young Scientists (B). Dr Okuya has received speaker's honoraria from Meiji and has received a Fujita Health University School of Medicine research grant, as well as a MEXT/JSPS KAKENHI Grant for Young Scientists. Dr Hatano has received speaker's honoraria from Dainippon Sumitomo and Otsuka. Dr Iwata has received speaker's honoraria from Dainippon Sumitomo, Eli Lilly, GlaxoSmithKline, Janssen, Yoshitomi, Otsuka, Takeda, Meiji, and Pfizer, along with research grants from Daiichi Sankyo, Takeda, Dainippon Sumitomo Eisai, Meiji, Tanabe‐Mitsubishi, and Otsuka.

## AUTHOR CONTRIBUTIONS

TK was involved in the study concept and design and performed the statistical analysis. TK, KS, MO, and MH performed acquisition and interpretation of the data. All the authors wrote the manuscript. NI supervised the review.

## INFORMED CONSENT

Informed consent was obtained via an opt‐out form on the website and in‐hospital bulletin board. Patients with unknown start times for LAI‐SGA therapy or who declined to participate were excluded.

## REGISTRY AND THE REGISTRATION NO. OF THE STUDY

n/a.

## ANIMAL STUDIES

n/a.

## ETHICAL APPROVAL

The study was approved by the Fujita Health University review board (HM20‐495) and was compliant with the Japanese Ethical Guidelines for Clinical Studies and the Declaration of Helsinki.

## Data Availability

The entirety of the patient's data cannot be made publicly available as data sharing was not included in the consent.
